# The association between prescribed hormonal contraception and multiple sclerosis risk: a systematic review and meta-analysis

**DOI:** 10.1007/s10072-026-08953-1

**Published:** 2026-03-13

**Authors:** Jessica McDonald, Priya Sharma, Christopher Tench, Ruth Dobson, Radu Tanasescu, Sonia Gran, Bruno Gran

**Affiliations:** 1 Mental Health and Clinical Neuroscience Unit, School of Medicine, University of Nottingham, Nottingham, UK; 2 Preventive Neurology Unit, Wolfson Institute of Population Health, Queen Mary University London, London, UK; 3 Department of Neurology, Nottingham University Hospitals NHS Trust, Nottingham, UK; 4 Lifespan and Population Health Unit, School of Medicine, University of Nottingham, Nottingham, UK

**Keywords:** Multiple sclerosis, Epidemiology, Hormonal contraception, Prevention

## Abstract

**Background:**

Sex hormones can influence susceptibility to multiple sclerosis (MS). Around 400 million women worldwide use hormonal contraceptives (HC), which contain synthetic versions of female sex hormones. Evidence on HC use and MS risk is inconclusive. The aim of this study was to synthesise studies on the association between prescribed HC and the risk of MS.

**Methods:**

Medline, Embase, Cochrane library and grey literature were systematically searched on 7th November 2024. Two reviewers independently screened, extracted data and appraised each study’s quality using the Joanna Briggs Institute critical appraisal tool. Pre-determined criteria were used to differentiate between high- and low-quality studies. Random-effects meta-analysis was conducted for studies providing the same effect measure; heterogeneity was assessed. Sensitivity analyses included only high-quality studies.

**Results:**

Eleven studies were included, six of these were cohort and five were case-control. Three studies were deemed high-quality. Five of the studies found no significant association between HC use and MS risk, four found a decreased risk, and two found an increased risk. Meta-analysis of the unadjusted odds ratio (OR) showed a significantly increased risk of MS with HC use of 1.28 (95% CI 1.08–1.52). However meta-analysis of adjusted ORs and sensitivity analyses showed no significant association. Heterogeneity was high.

**Conclusion:**

No statistically significant association between HC use and MS risk was observed. The meta-analyses indicate a possible increased risk warranting further investigation. Given that heterogeneity was high and only three of the included studies were classed as high-quality, a well-designed study is needed to clarify this association.

## Introduction

Multiple Sclerosis (MS) is a chronic inflammatory disease of the central nervous system (CNS) characterised by immune-mediated demyelination and axonal loss [1]. It is the leading cause of non-traumatic neurological disability in young adults in high-prevalence countries (including Europe, North America, Australia, and New Zealand), with a mean age of onset of 20–30 years [1, 2]. The cause of MS is thought to be multifactorial with both genetic and environmental risk factors playing key roles. Current known environmental risk factors include infection by Epstein-Barr virus (EBV), smoking, low serum levels of vitamin D, and childhood obesity [1]. Over 200 gene variants also contribute to MS risk, many related to immune system functioning [3].

Gender differences in the incidence rate and disease progression of MS suggest that sex hormones, such as oestrogens, progesterone and androgens, may play a role in disease course and susceptibility. MS is 2–3 times more prevalent in women than men, and the sex ratio appears to be increasing [4]. Women tend to experience more frequent relapses, whereas disease progression tends to be faster in men [5].

Oestrogens’ effects on the immune system have been shown to depend on hormone concentration, with induction of proinflammatory cytokines at lower concentrations and induction of T-helper 2 (anti-inflammatory) cytokines at higher concentrations [6]. This is further supported by research into the protective effects of pregnancy, which is associated with high oestrogen and progesterone levels, with studies reporting a 70% decrease in MS relapse rates during the third trimester compared to pre-pregnancy levels [7]. Progesterone has also been shown to have neuroprotective, promyelinating and anti-inflammatory effects in the CNS [8].

Hormonal contraceptives (HC) are available in various formulations, primarily combining synthetic oestrogens with progestins, or containing progestins alone [9]. Oral contraceptives are most commonly used, with alternatives including injectable progestins, hormonal intrauterine devices, transdermal patches and vaginal rings [10, 11]. The pharmacokinetics, hormonal dosage and routes of administration vary across these formulations [12]. These variations may influence inflammatory and immune pathways differently, raising the possibility that specific HC types may differentially impact MS risk.

With substantial evidence suggesting a major role of sex hormones in the regulation of MS disease course and inflammation, paired with possible neuroprotective benefits, HC has the potential to influence MS susceptibility and clinical course. Given that around 400 million women worldwide use HC [13], and the female predominance of MS is increasing, it is clinically important to determine any association. Understanding this relationship will be essential for informing contraceptive decision making, particularly among individuals at an increased risk of MS, guiding future research efforts in sex hormones and MS progression, and the potential utility of hormonal therapies for disease prevention or management. Alternatively, clear evidence demonstrating no association would provide reassurance to women that HC use will not influence their risk of developing MS.

To address this important research gap, we conducted a systematic review and meta-analysis investigating the relationship between prescribed HC use and the risk of developing MS. To our knowledge, this is the first systematic review and meta-analysis specifically examining this association.

## Methods

### Protocol registration

The protocol for this systematic review and meta-analysis was registered on PROSPERO on 15th October 2024 (Registration number: CRD42024601415). This study followed the Preferred Reporting Items for Systematic Review and Meta-Analyses (PRISMA) guidelines.

## Literature search strategy

The databases searched included MEDLINE and Embase, via Ovid, as well as the Cochrane library.

An information specialist from the University of Nottingham was consulted when designing and finalising the search strategy to ensure the search was thorough. The exact search strategy used for this review can be found in Appendix A.

The final searches were conducted on 7th November 2024.

The titles and abstracts were exported from MEDLINE, Embase and Cochrane library into Rayyan software. Potential duplicates were identified by Rayyan and resolved by the researcher. Grey literature was also searched using ProQuest Dissertations & Theses. Additionally, retrospective hand searching of the reference lists of the articles identified after full text screening was conducted to ensure relevant studies weren’t missed.

## Inclusion and exclusion criteria and screening process

The population consisted of women of any age, with no restrictions on geographic locations, ethnicities or language of the study. The exposure was prescribed HC medication. There were three comparators: (i) women who have never been exposed to HC, (ii) women who did not use HC during the study period but may have done so previously and (iii) women who take different types of HC, to determine which medication is most associated with MS. The outcome assessed was a diagnosis of MS. We included observational studies (Cohort, Cross-sectional, Case-Control, Case series with > 30 participants, for hypothesis purposes), and randomised controlled trials. A full list of the inclusion and exclusion criteria used for study selection can be found in Appendix B. In the protocol, the population originally consisted of women over the age of 18, however this was changed to include all women from age 12 onwards. This change was made to ensure that any relevant studies were not excluded.

The online tool Rayyan was used for title and abstract, and full text screening. All screening stages were conducted independently by at least two reviewers (JM, PS, and BG).

## Quality assessment

Following full text screening, all studies identified to be included were then critically appraised to assess the quality of the papers. All included studies were quality assessed by at least two reviewers independently (JM, PS, and BG).

The risk of bias was assessed using the Joanna Briggs Institute critical appraisal checklists, with an additional custom question regarding the type of HC used [14].

Studies were classed as high-quality based on the following criteria:

1. Outcome and exposures measured in a reliable way.
2. Type of hormonal contraception considered in analysis.
3. Analysis has adjusted for important confounding variables (e.g. body mass index or confounding by indication).

In the protocol there was a fourth criteria point for classifying studies as high-quality which was, ‘Prospectively collected data source used’. However, after initial review, it was decided to remove this as it was difficult to consistently determine for most of the included papers.

## Data extraction

A data extraction table was created in Microsoft Excel based on the information in the protocol. A list of the information that was extracted from the studies can be found in Appendix C.

Data extraction was completed by the primary researcher for all the included studies (JM). A clinical reviewer (PS) completed 10% of the data extraction independently, which amounted to two studies. These two studies completed by PS were identified by the primary researcher as being the most difficult to extract.

### Meta-analysis

Meta-analyses were conducted using Cochrane’s Review Manager version 5.4 software. Meta-analyses could only be conducted for studies which had the same effect measure. The effect measure provided most often by the included studies was used.

The following meta-analyses with random effects models and inverse-variance weighting were conducted:

- Unadjusted ORs.
- Adjusted ORs.

Sensitivity analyses of both the unadjusted and adjusted ORs were also conducted. These consisted of only the studies deemed high-quality.

Heterogeneity was assessed through I^2^ as well as visually comparing the 95% confidence intervals (CI) in the forest plots.

Subgroup analysis was planned to explore heterogeneity (see protocol for further detail). However, due to the small number of included studies and lack of data provided by these studies, this was not possible.

## Results

### Literature search

Searches revealed 3,041 studies; duplicates were identified and resolved by Rayyan software leaving 2,182 titles and abstracts to be screened.

**Fig. 1:**
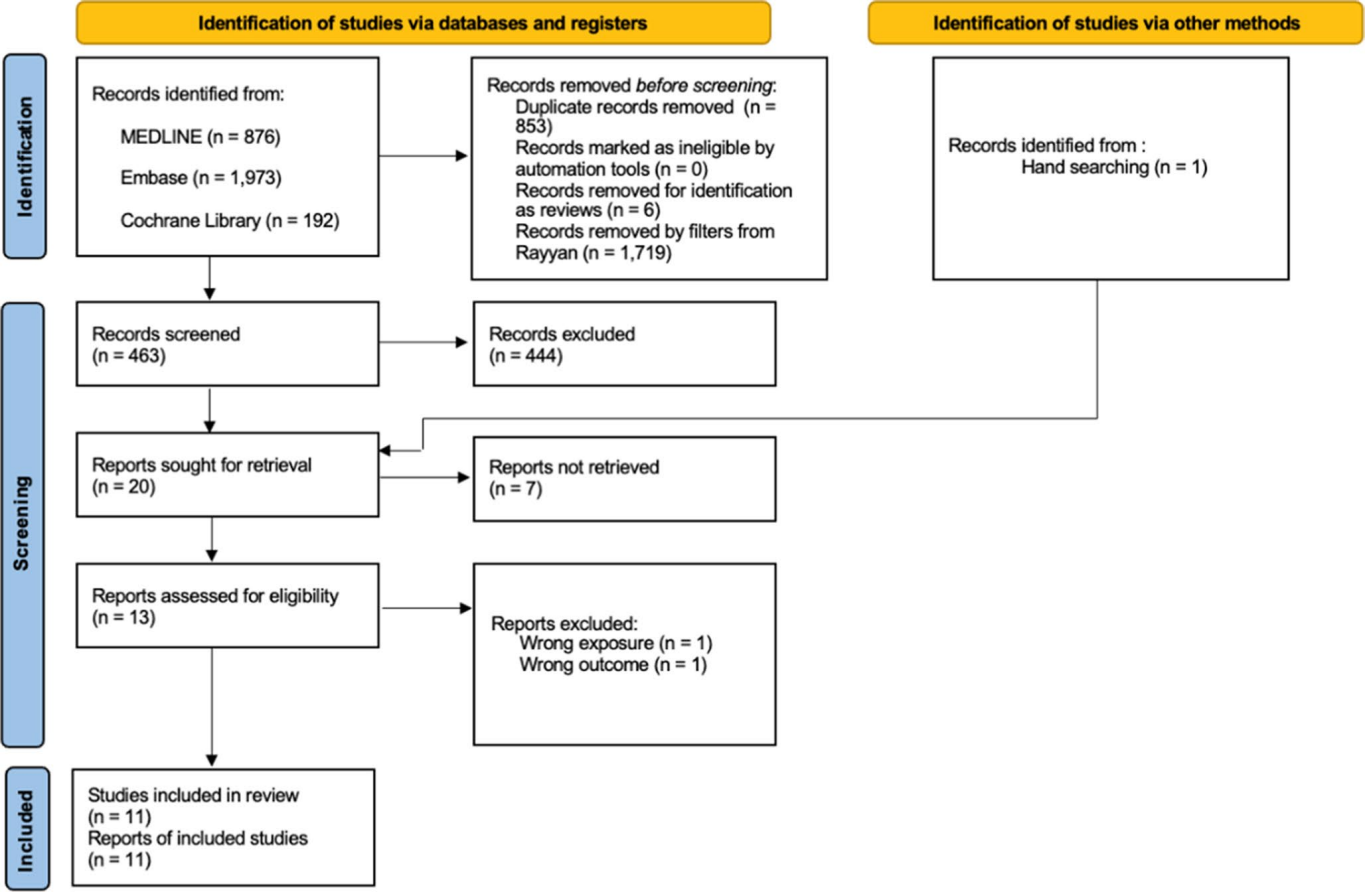
PRISMA flow diagram, showing the number of studies included and excluded at each stage of the systematic review

Following title and abstract screening, and retrospective hand searching through the references, 20 articles were identified for retrieval of full texts. Of the 20 articles, 13 were obtained, of which two were excluded because of wrong exposure and wrong outcome (see Fig. 1). Seven studies were either too old or not available as full text to be obtained.

### PRISMA diagram

The PRISMA flow diagram (Fig. 1) shows the number of studies at each stage of the systematic review [15].

### Overview of included studies

Eleven studies were included in the systematic review. Details of these studies are shown in Table 1. All the studies were in English. Six were cohort studies and five were case-control studies. Five studies were from the UK, three from Iran, two from the USA, and one from Sweden.

**Table 1:** Studies included

Primary author; publication year	Study design	Geographic location	Primary objective	Study duration and follow-up period	Number of participants	Type of control group	Number of women in control group	Number of MS cases	How outcome was measured	Type of HC investigated	How exposure was measured	Confounders adjusted for	Clinical interpretation summary	Quality of study
A.Alonso; 2005	Case-control	UK	Examine whether recent OC use and pregnancy history are associated with risk of MS	10 years	1,107	Women without MS Controls matched on age, general practice and date of joining practice	1,001 (mean age is 33.7+/−8.0)	106 (mean age is 33.4+/−8.1)	MS diagnosis made using International Classification of Diseases, ninth revision)	OC pill	Information regarding OC use obtained from computerised medical records in the general practice research database	Age, general practice, date of joining practice, smoking status and BMI	Recent OC use associated with 40% reduction in MS incidence, therefore suggesting protective effect	Low
K.Hellwig; 2016	Case-control	Southern California	Determine whether there is an association of COC use and risk of developing MS/CIS	4-year duration, 10 year follow up	4,304	Women without MS. Controls matched on age, race/ethnicity and KPSC membership characteristics	3904 (mean age is 33.8+/−9.2)	400 (mean age is 33.8+/−9.2)	MS diagnosed by MS specialist according to revised McDonald criteria	COCP and sub-group analysis stratified by progestin content	Pharmacy records within 10 years of index date obtained to find out COCP use status	BMI, smoking, live births and abortions/miscarriages	COCP use associated with a slight increased risk of MS/CIS	High
M.A.Hernán; 2000	Cohort	U. S	Determine whether there is an association between COCP use and incidence of MS	18 years (NHS) and 8 years (NHS II)	121,700 (NHS) 116,671 (NHS II)	Women not exposed to OC	64,220 (NHS) 19,244 (NHS II)	181 MS cases during follow up (NHS) 134 MS cases during 6 years follow up (NHS II)	Relevant medical records from participants who reported MS diagnosis during follow up were obtained and Poser criteria for MS diagnosis applied o clinical and laboratory data obtained	COCP	Information regarding COCP use obtained by questionnaire sent to participants. This information was updated every 2 years	Age, latitude tier (north, middle, south), ancestry and smoking status	Findings do not support the e existence of a strong association between COCP use and incidence of MS	Low
P.Holmqvist; 2010	Cohort	Sweden	Determine whether there is a relation between age of onset of MS and use of COCP and/or timing of childbirth	unclear	770	Women not exposed to OC	unclear	770	MS cases identified from Swedish Multiple Sclerosis (SMS) Register	OC pill	Information regarding OC use obtained from questionnaire. This information was checked against existing data in SMS register	Duration of OC use, number of childbirths before MS onset, age at OC initiation	Mean age of MS onset is higher with longer duration of OC use, suggesting a protective effect	Low
A.Mohammadbeigi; 2016	Cohort	Iran (Isfahan)	Investigate the risk factors of early inset of MS in women during reproductive years	unclear	200 (mean age is 31.77+/−8.13)	Women not exposed to HC medications	unclear	200	MS diagnosis confirmed by diagnostic studies (MRI) and approved by neurology committee of hospital using standard McDonald criteria	OC pill	Information regarding OC use obtained by interview	Education level, marital status and parity	Significantly higher age of onset of MS in ever users of OC compared to never users	Low
A.Nova; 2024	Cohort	UK	Determine whether there is an association between OC pills and risk of developing MS	Median follow up for 1 st analysis: 71years Median follow up for 2nd analysis: 35years	181,058	Women not exposed to HC medications	32,098	1,131	MS diagnosed using International Classification of Disease, 10th revision diagnosis code G35	OC pill	Information regarding OC use obtained by questionnaire	MS polygenic risk score, education level, parity, smoking, fertility problems, obesity and mononucleosis	No significant association between OC use and MS risk found. However, 30% increase in lifetime MS hazard for ever OC users and 35% increase in MS hazard for current OC users	Low
M.Rejali; 2016	Case-control	Iran (Isfahan)	To evaluate some of the risk factors for MS in women of childbearing age	unclear	400	Women without MS Controls matched according to area of residence	200 (mean age is 31.53+/−8.97	200 (mean age is 31.76+/−8.13)	MS diagnosed by neurology committee of hospital with use of diagnostic studies such as MRI	OC pill	Information regarding OC use obtained by interview	Age, marital status, place of residence, family history of MS, other autoimmune diseases and history of childhood viral diseases	Use of OC and longer duration of use associated with reduced risk of MS	Low
F.Salehi; 2018	Case-control	Iran	Determine whether there is an association between females’ reproductive age-related factors and MS risk	unclear	940	Women without MS	541 (mean age is 31.73+/−9.02)	399 (mean age is 30.64+/−7.58)	MS diagnosis based on 2010 McDonalds criteria along with positive MRI scan	OC pill	Information regarding OC use obtained by interview	Age, marital status, education and ethnicity	Use of OC associated with increased risk of MS. Lower age at first OC use further increases risk of MS	Low
M.Thorogood; 1998	Cohort	UK	Examine the risk of MS in users of COCP	28 years	46,000	Women not exposed to OC	Around 23,000 (not entirely clear)	114	MS diagnosis reported by GP using International Classification of Disease, 8th revision	COCP (subgroup analysis stratified by oestrogen content) and POP	Information regarding OC use obtained from GP records?	Age, parity, social class and smoking	No significant increased risk of MS found in COCP users. Slightly higher risk of MS associated with COCP use containing higher doses of oestrogen	High
L.Villard-Mackintosh; 1993	Cohort	UK (Oxford)	Examine effects of pregnancy, parity and OC use on risk of MS development	unclear	17,032	Women not exposed to OC	unclear	63	MS diagnosed using International Classification of Disease 8th revision	COCP	unclear	Age, parity and smoking	COCP use associated with slightly lower rate of onset of MS than non-users	Low
Q.Zhang; 2024	Case-control	UK (East London)	Determine whether there is an association of prior OC use with subsequent MS	28 years (1990–2018)	4,455	Women without MS Controls matched by birth year and month	3,564 (mean age is 47+/−14.3)	891 (mean age is 47+/−14.3)	MS cases identified through electronic primary care data in East London	COCP and POP	COCP and POP prescription information identified through electronic primary care data in East London	Age, ethnicity and index of multiple deprivation	No significant association between OC use and MS risk	High

Abbreviations: *HC* hormonal contraceptive, *OC* oral contraceptive, *MS* Multiple Sclerosis, *BMI* body mass index, *COC* combined oral contraceptive, *CIS* clinically isolated syndrome, *KPSC* Kaiser Permanente Southern California, *COCP* combined oral contraceptive pill, *NHS* Nurses’ Health Study, *NHS II* Nurses’ Health Study II, *SMS Register* Swedish Multiple Sclerosis Register, *GP* general practitioner, *POP* Progestin only pill

### Measurement of outcome

Four of the studies used the International Classification of Disease (ICD) to define MS cases. Villard-Mackintosh et al. and Thorogood et al. used ICD-8 [16, 17], Alonso et al. used ICD-9 [18], and Nova et al. used ICD-10 [19]. Three studies used the McDonald criteria. Salehi et al. used the 2010 revisions [20]. The other two, Mohammabeigi et al. and Hellwig et al., did not specify which revision was used [21, 22]. Hernán et al. used the Poser criteria [23]. Holmqvist et al. used the Swedish Multiple Sclerosis register for identification of cases [24]. Rejali et al. had MS diagnosed by neurology committee of local hospital following diagnostic studies [25]. Zhang et al. used existing electronic primary care data for MS identification [26].

### Quality assessment

Quality assessment for cohort and case-control studies is shown in Tables 2 and 3. Three studies were identified as high-quality studies according to the criteria previously stated in the methods Sects [22, 26, 27]. Two of these high-quality studies were case-control studies [22, 26], and one was a cohort study [27]. Eight studies were classified as low-quality [18–27]. Two of these eight studies were only deemed as low-quality due to failure to consider type of HC in analysis, but their methodology was otherwise good [18, 23].

**Table 2:** Quality assessment table for cohort studies. The study authored by L. Villard-Mackintosh was reclassified from case-control study to cohort study upon review of methodology

Primary author; Publication year	Were the two groups similar and recruited from the same population?	Were the exposures measured similarly to assign people to both exposed and unexposed groups?	Was the exposure measured in a valid and reliable way?	Were confounding factors identified?	Were strategies to deal with confounding factors stated?	Were the groups/participants free of the outcome at the start of the study (or at the moment of exposure)?	Were the outcomes measured in a valid and reliable way?	Was the follow up time reported and sufficient to be long enough for outcomes to occur?	Was follow up complete, and if not, were the reasons to loss to follow up described and explore?	Were strategies to address incomplete follow up utilised?	Was appropriate statistical analysis used?	Was type of hormonal contraceptive considered in analysis?	Overall quality of study
M.A. Hernán; 2000	yes	yes	yes	yes	yes	yes	yes	yes	unclear	unclear	yes	no	low
P. Holmqvist; 2010	yes	yes	yes	partially	yes	yes	unclear	no	unclear	unclear	yes	no	low
A. Mohammadbeigie; 2016	yes	yes	partially	yes	yes	no	unclear	unclear	unclear	unclear	yes	no	low
A. Nova; 2024	unclear	unclear	no	yes	yes	yes	yes	yes	no	no	yes	no	low
M. Thorogood; 1998	yes	yes	yes	yes	yes	yes	yes	yes	yes	yes	yes	yes	high
L. Villard-Mackintosh; 1993	yes	unclear	unclear	partially	unclear	unclear	yes	unclear	unclear	unclear	unclear	no	low

**Table 3:** Quality assessment table for case-control studies

Primary author; publication year	Were the groups comparable other than the presence of disease in cases or the absence of disease in controls?	Were the cases and controls matched appropriately?	Were the same criteria used for identification of cases and controls?	Was the exposure measured in a standard, valid and reliable way?	Was exposure measured in the same way for cases and controls?	Were confounding factors identified?	Were strategies to deal with confounding factors stated?	Were outcomes assessed in a standard, valid and reliable way for cases and controls?	Was the exposure period of interest long enough to be meaningful?	Was appropriate statistical analysis used?	Was the type of hormonal contraceptive considered in analysis?	Overall quality of study
A. Alonso; 2005	yes	yes	yes	yes	yes	yes	yes	yes	yes	yes	partial	low
K. Hellwig; 2016	yes	yes	yes	yes	yes	yes	yes	yes	yes	yes	yes	high
M. Rejali; 2016	yes	no	unclear	unclear	yes	yes	yes	yes	unclear	yes	no	low
F. Salehi; 2018	unclear	no	unclear	yes	yes	yes	yes	yes	unclear	no	no	low
Q. Zhang; 2024	yes	yes	yes	yes	yes	yes	yes	yes	yes	yes	yes	high

Four of the studies had unreliable or unclear methods of assessing exposure [16, 19, 21, 25]. One study did not state whether confounders had been adjusted for or not [16]. Two studies had unreliable or unclear methods of assessing outcome [21, 24]. Eight studies did not consider the type of HC used in analysis [18–27].

Upon review of the methodology described by each included paper, one of the studies, namely the study authored by L. Villard-Mackintosh, was reclassified from a case-control study to a cohort study [16]. This was to ensure the appropriate JBI checklist could be used for critical appraisal.

### Meta-analysis

Five of the 11 studies were included in meta-analysis as they had effect measures that could be pooled [18, 20, 22, 25, 26]. Subgroup analyses could not be conducted due to insufficient comparison points across studies:

- All five studies included in the meta-analysis were case-control studies and therefore comparison between different study types was not possible [18, 20, 22, 25, 26].
- Two of the studies provide results regarding the onset of HC use and MS risk, however one of these is unadjusted [20] and the other is adjusted [25].
- Three of the studies give results on duration of HC use. One of these studies uses different duration categories to the others (less than 2 years, 2–5 years, and over 5 years [26] compared to less than 12 months and over 12 months [18, 20]). For the two studies that use the same duration categories, one provides an unadjusted OR [20], whereas the other provides an adjusted OR [18].

Similarly, comparisons between different HC types were not feasible, as only one of the five studies included in meta-analysis provided separate information on HC type used in the analysis [26].

### Unadjusted OR meta-analyses

**Fig. 2:**
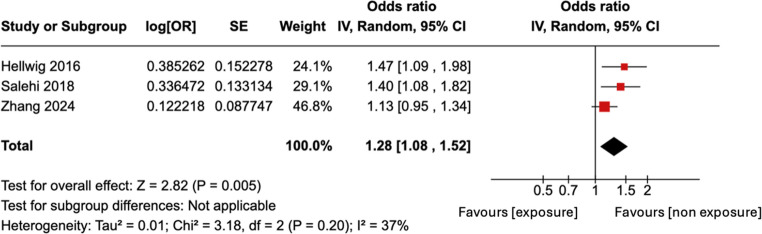
Forest plot for meta-analysis of unadjusted ORs for developing MS. The OR and its 95% CI is to the right of the vertical line (i.e. greater than 1), indicating a statistically significantly increased risk of MS associated with HC use

The pooled unadjusted OR meta-analysis consists of 3 of the 5 included studies as only these 3 studies provided unadjusted ORs [13, 14, 19]. This meta-analysis showed a statistically significant OR of 1.28, with 95% CI of 1.08–1.52 (Fig. 2). This suggests a 28% increased risk of developing MS following HC use. The I^2^ index is 37% showing moderate heterogeneity.

**Fig. 3:**
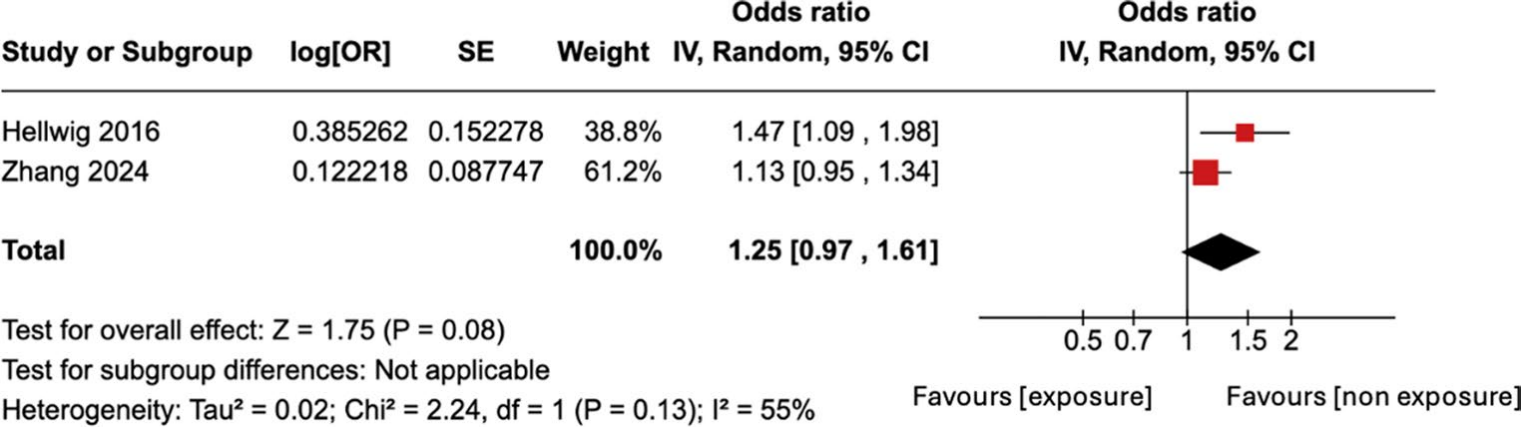
Forest plot for meta-analysis of unadjusted ORs for developing MS, for the studies classified as high-quality. The OR is to the right of the vertical line (i.e. greater than 1), and there is a trend (*p* = 0.08) towards significantly increased risk of MS associated with OC use

However, the sensitivity analyses (including only high-quality studies with unadjusted ORs) found no statistically significant association with a pooled OR of 1.25 and 95% CI of 0.97–1.61 (Fig. 3). This suggests that the initial meta-analysis result may be due to bias associated with the included low-quality study. The I^2^ index was 55%, showing moderate heterogeneity.

### Adjusted OR meta-analyses

The adjusted OR meta-analysis consisted of all 5 included studies as they all provided adjusted ORs. The confounders adjusted for by each study in the meta-analysis can be found in Table 4.

**Table 4:** Confounding variables, and which studies adjusted for each confounder in analysis. The numbers refer to the bibliography

Confounding variables	Studies that adjusted for this confounder
BMI	(13, 18)
Smoking status	(13, 18)
Live births	(13)
Abortions and/or miscarriages	(13)
Age	(14, 17, 19)
Marital status	(14, 17)
Education level	(14)
Ethnicity	(14, 19)
Index of multiple deprivation	(19)
Place of residence	(17)
Family history of MS	(17)
Presence of other autoimmune disorders	(17)
History of childhood viral disease	(17)

Abbreviations: *BMI* Body mass index, *MS* Multiple Sclerosis

**Fig. 4:**
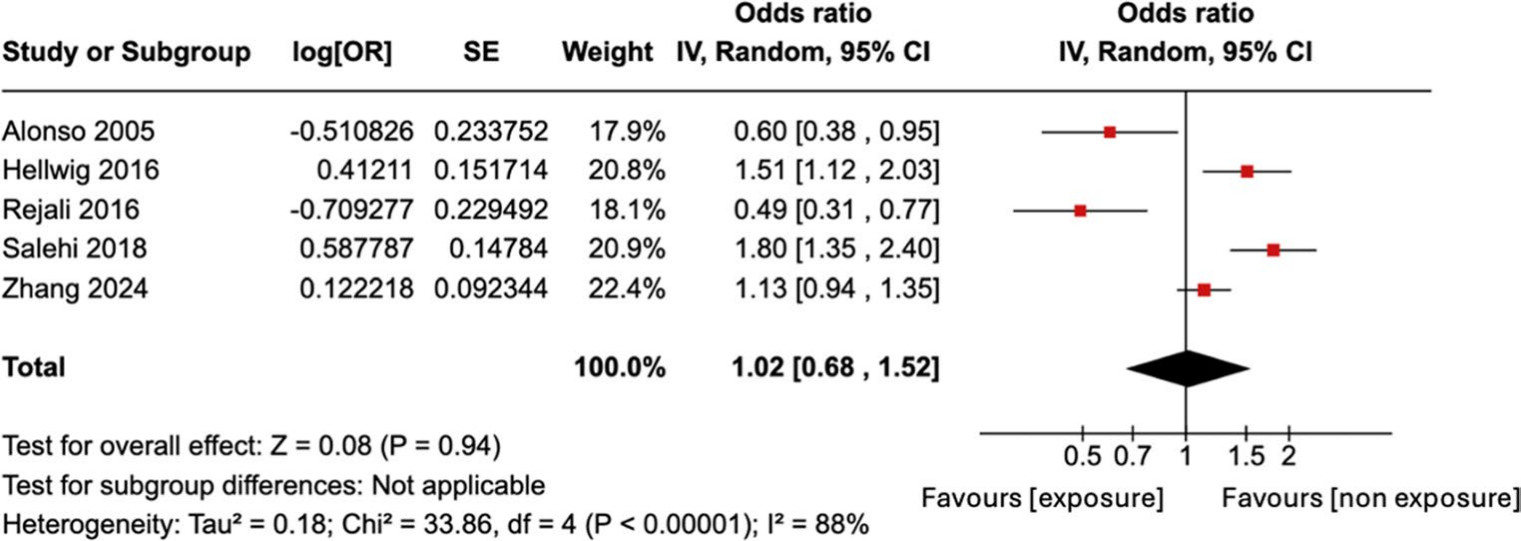
Forest plot for meta-analysis of adjusted ORs for developing MS. There is evident heterogeneity

This meta-analysis found no statistically significant result with a pooled OR of 1.02 and 95% CI of 0.68–1.52 (Fig. 4). The I^2^ index is 88% which shows considerable heterogeneity.

**Fig. 5:**
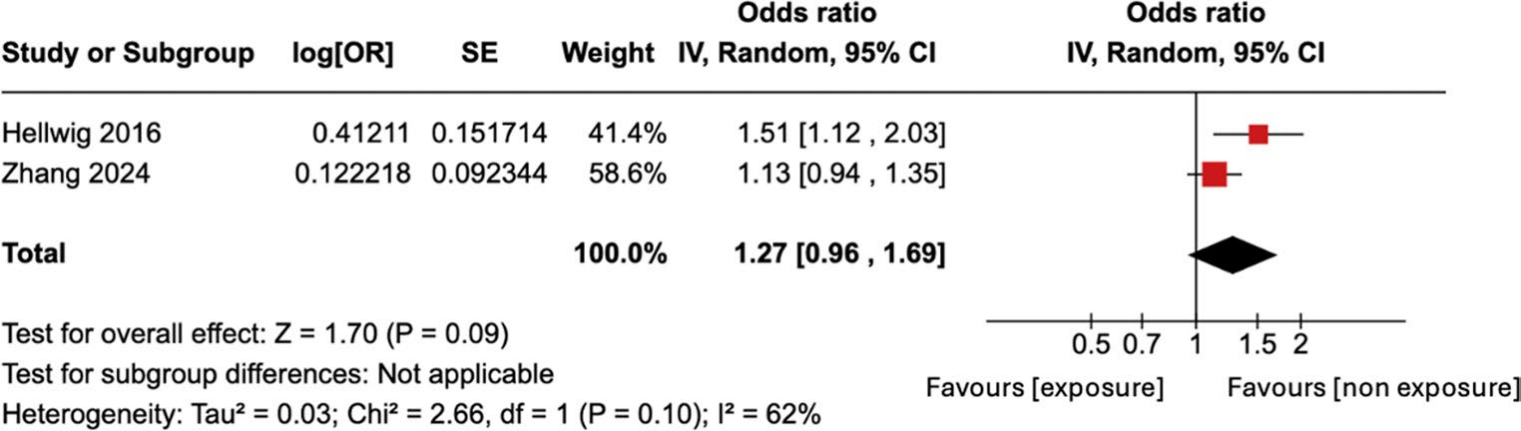
Forest plot for meta-analysis of adjusted ORs for developing MS, for the studies classified as high-quality. The OR is to the right of the vertical line (i.e. greater than 1) but is not statistically significant (*p* = 0.1)

The sensitivity analysis (including only high-quality studies with adjusted odds ratios) also found no statistically significant result with a pooled adjusted OR of 1.27 and 95% CI of 0.96–1.69 (Fig. 5). The I^2^ index was 62% which suggests substantial heterogeneity.

### Narrative analysis

Narrative analysis of the 11 included studies further demonstrates inconsistent findings.

Five of the included studies showed no significant association between the use of HC and the risk of developing MS [16, 19, 23, 26, 27]. Two of these, Zhang et al. and Thorogood et al., were classified as high-quality studies [26, 27]. Both of these studies also investigated the separate effects of the use of the combined oral contraceptive (COC) pill and the progesterone only pill on MS risk, but found no significant association. A large study using the UK Biobank suggested that parity acts as a modifier for the association between HC use and the risk of MS development, as in nulliparous women the hazard ratio for MS risk was higher in current HC users compared to ever HC users [19]. This study used patient questionnaires, introducing a risk of recall bias.

Two of the included studies showed an increased risk of MS development with HC use [20, 22]. However, in Hellwig et al.’s study the risk of MS did not vary with duration of HC use [22].

The remaining four studies showed a decreased risk of MS with HC use [18, 21, 24, 25]. Two of these studies assessed the association between oral contraceptive (OC) use and age of onset of MS. Holmqvist et al. found the mean age of MS onset in COC users to be 26 years compared to 19 years in non-users (*p* < 0.001) [24]. Mohammadbeigi et al. found the mean age of MS onset in OC users to be 30.2 (95% CI 28.4–32.0) compared to 25 (95% CI 23.75–26.25) in never-users (*p* < 0.001) [21]. Rejali et al. found a decreased risk of MS with prior OC use, giving an adjusted OR of 0.492 (95% CI 0.314–0.772, *p* = 0.002). Furthermore, Rejali et al. found this decreased risk to be enhanced with longer duration of OC use, adjusted OR was 0.881 (95% CI 0.803–0.967, *p* = 0.008) [25]. Finally, Alonso et al. found MS incidence to be 40% lower in OC users compared to non-users, with an adjusted OR of 0.6 (95% CI 0.4–1.0.4.0) [18].

Only three of the studies discussed how different preparations of HC may affect the risk of MS. Hellwig et al. found that the association between COC use and MS risk varied depending on the progestin content with preparations containing levonorgestrel giving a stronger association (adjusted OR = 1.75, 95% CI 1.29–2.37, *p* < 0.001) than preparations containing norethindrone (adjusted OR = 1.57, 95% CI 1.16–2.12, *p* = 0.003) and no association for those containing drospirenone (*p* = 0.95) [22]. Thorogood et al. suggested that there may be a higher risk of MS for users of COC preparations containing higher doses of oestrogen (≥ 50 micrograms of oestrogen), although this finding was not statistically significant and was limited by small sample size [17]. Zhang et al. found no significant association with either COC or progesterone only HC types and MS risk [26].

Nine studies investigated how duration of HC use impacted the risk of MS. Of these, six demonstrated no clear trend [16–18, 20, 22, 23], one reported an increased risk of MS for longer HC duration, but only in nulliparous women [19], and two studies observed a reduced risk with increasing duration [24, 25]. As discussed previously, Hellwig et al. found a statistically significant increased risk of MS/CIS with COC use; however, this risk did not change with increasing duration of COC use, which is a strong indicator of a non-causal relationship [22]. Similarly, Salehi et al. found an overall increased risk of MS with HC users, but no significant association between duration of use and MS risk. Alonso et a. observed an approximate 40% reduction in MS incidence with OC use, yet likewise reported no clear duration dependent trend. In contrast Rejali et al. and Holmqvist et al. both identified a protective link between HC use and MS risk, with findings suggesting greater risk reduction or later onset of disease among women with longer durations of use [24, 25].

Five studies found no association between time since last HC use and MS risk [16–18, 23, 26]. Four studies investigated the effects of age at onset of HC use and MS risk, two of these found that earlier age of HC onset was associated with increased risk, however one of these studies, Nova et al., only found this to be true for nulliparous women. The remaining two studies found no such trends [21, 25].

## Discussion

This systematic review synthesised published data on the possible association between HC and MS risk. The topic is important as MS is 2–3 times more frequent in women and the mechanisms underpinning such difference are incompletely understood. The main findings indicate that such association remains unclear. A large, well-designed epidemiological observational study is required to fill this important research gap.

Our unadjusted OR meta-analysis consisted of three studies (two of high and one of low quality). The pooled results showed a statistically significant increase in the risk of developing MS with HC use by 28% (95% CI 1.08–1.52).

This finding is supported, in part, by those of a retrospective study using British National Health Service data to analyse the risk of MS in individuals with gender identity disorder (GID). This study, conducted by Pakpoor et al., found a significantly elevated adjusted relative risk for MS of 6.63 (95% CI 1.81–17.01) following a diagnosis of GID in men; no such association was found in women diagnosed with GID [28]. These data suggest that female gender affirming treatment, which typically consists of exogenous oestrogens or testosterone suppression, may be associated with an increased risk of MS [29]. This would support the findings of our meta-analysis, suggesting that oestrogens found in OC medications could increase the risk of MS. However, there were several limitations to that retrospective study including a small sample size of patients with GID and a lack of detail about the gender affirming treatment used [28, 29].

The sensitivity analysis for the two high-quality studies [22, 26] providing unadjusted ORs showed no significant association, suggesting that the initial meta-analysis result may be due to biases associated with the included low-quality study [20].

Our adjusted OR meta-analysis across five studies showed no significant results with a pooled OR of 1.02 (95% CI 0.68–1.52). The lack of association emphasises the importance of considering confounders (Table 4), which may substantially impact findings.

### Strengths and limitations

This systematic review has several methodological strengths. A comprehensive and sensitive search strategy was developed, ensuring the inclusion of all relevant studies. Importantly, there were no restrictions on publication language, geographic location or date of publication. This both reduced the risk of selection bias and ensured that no relevant studies would be missed. In addition, retrospective hand searching through references of select studies further ensured, to the best of our ability, that no relevant studies were excluded.

The main limitations of this review stem from the limited number of relevant studies available in the literature. This underscores the need for further research into this important aspect of MS susceptibility. Many of the studies were retrospective in design, increasing the risk of recall and selection bias. Furthermore, confounding by indication, BMI, parity and other important variables were inconsistently accounted for, resulting in considerable heterogeneity between the included studies. The studies included in this review had methodological limitations, notably the lack of consideration of the type of HC used in the analyses resulting in ambiguity for what type of HC is being used such as COC vs. progesterone only. None of the included studies provided data about the use of non-oral forms of HC such as the subdermal implant, vaginal ring, or intra-uterine system [19–27].

Another limitation included the high heterogeneity across included studies, substantially minimising the reliability and interpretability of the pooled estimates [30]. Inconsistent adjustment for confounders (Table 4) is likely to contribute to this heterogeneity, further limiting comparability between studies. Outcome measurement also differed across studies. Although eight of the studies used MS diagnosis as the primary outcome, varying diagnostic criteria were used, further details into this are discussed in the results section of this article [16–20, 23, 25, 26]. Two studies examined the age at MS onset rather than incidence [21, 24], and one study included both MS and CIS in the outcome [22]. These methodological differences further limit comparability and highlight the need for cautious interpretation of the pooled findings.

## Conclusion

This systematic review and meta-analysis highlights inconsistent findings across existing studies evaluating the association between HC use and the risk of MS, identifying a gap in the current literature. Interpretation of the pooled estimates is limited by substantial heterogeneity and the small number of included studies, which reduce the reliability of the overall effect estimates. As such, current evidence remains insufficient to draw conclusions regarding any association between HC use and MS risk. Further research into this association is warranted and may help to explain the increasing sex ratio. A large, well-designed, prospective population-based case-control study including users of all types of HC, adjusting for important confounders, and exploring a dose-response relationship is required. Such research could have significant clinical relevance. If an association between HC use and increased MS risk exists, individuals at higher risk of MS could explore the use of other contraceptive methods. Alternatively, if a protective link between HC use and MS risk is established, this could lead to further research into the use of hormonal therapies to prevent MS. Finally, if no association is found, the results could be used to more conclusively reassure women at higher risk of MS that they can safely use HC.

## Data Availability

Available upon request.

## Appendix A: MEDLINE, embase and cochrane library search strategy

The search strategy for MEDLINE was:

1. exp Multiple Sclerosis/
2. “multiple sclerosis”.mp.
3. MS.ti,ab.
4. (“clinically isolated syndrome” or “first demyelinating event”).mp.
5. (“radiologically isolated syndrome” or “incidental MRI findings” or “incidental demyelination”).mp.
6. 1 or 2 or 3 or 4 or 5
7. Hormonal Contraception/
8. exp Contraceptives, Oral/
9. Intrauterine Devices, Medicated/
10. ((“intra-uterine device*” or “intrauterine device*” or iud*) adj4 (medicated or hormonal or “hormone-releasing” or “progesterone-releasing”)).mp.
11. exp Ethinyl Estradiol/
12. (“ethinyl estradiol” or ethinylestradiol or “ethinyl oestradiol” or ethinyloestradiol).mp.
13. Desogestrel/
14. Levonorgestrel/
15. Norethindrone Acetate/
16. ((progestin* or progesterone) adj4 contracept*).mp.
17. (“medroxyprogesterone acetate” adj4 contracept*).mp.
18. Contraceptives, Postcoital/
19. ((oral* or patch* or inject* or hormo* or medicated or postcoit* or “post coit*”) adj3 contracept*).mp.
20. (estriol or oestriol).mp.
21. 7 or 8 or 9 or 10 or 11 or 12 or 13 or 14 or 15 or 16 or 17 or 18 or 19 or 20
22. 6 and 21

The search strategy for Embase was:

1. exp multiple sclerosis/
2. “multiple sclerosis”.mp.
3. (“clinically isolated syndrome” OR “first demyelinating event”).mp.
4. (“radiologically isolated syndrome” OR “incidental MRI findings” OR “incidental demyelination”).mp.
5. MS.ti,ab.
6. 1 or 2 or 3 or 4 or 5
7. hormonal contraception/
8. exp oral contraceptive agent/
9. intrauterine contraceptive device/
10. levonorgestrel releasing intrauterine system/
11. ((“intra-uterine device*” or “intrauterine device*” or iud*) adj4 (medicated or hormonal or “hormone-releasing” or “progesterone-releasing”)).mp.
12. ethinylestradiol/
13. (“ethinyl estradiol” or ethinylestradiol or “ethinyl oestradiol” or ethinyloestradiol).mp.
14. desogestrel/
15. levonorgestrel/
16. norethisterone acetate/
17. ((progestin* or progesterone) adj4 contracept*).mp.
18. (“medroxyprogesterone acetate” adj4 contracept*).mp.
19. exp postcoitus contraceptive agent/
20. ((oral* or patch* or inject* or hormon* or medicated or postcoit* or “post coit*”) adj3 contracept*).mp.
21. (estriol OR oestriol).mp.
22. 7 or 8 or 9 or 10 or 11 or 12 or 13 or 14 or 15 or 16 or 17 or 18 or 19 or 20 or 21
23. 6 and 22

The search strategy for Cochrane Library was:

1. MeSH descriptor: [Multiple Sclerosis] explode all trees
2. “multiple sclerosis”
3. (MS):ti OR (MS):ab
4. “clinically isolated syndrome”
5. “first demyelinating event”
6. “radiologically isolated syndrome”
7. (“incidental MRI findings” or “incidental demyelination”)
8. #1 or #2 or #3 or #4 or #5 or #6 or #7
9. MeSH descriptor: [Hormonal Contraception] this term only
10. MeSH descriptor: [Contraceptives, Oral] explode all trees
11. MeSH descriptor: [Intrauterine Devices, Medicated] this term only
12. MeSH descriptor: [Contraceptives, Postcoital] explode all trees
13. (“intra-uterine device” or “intra-uterine devices” or “intrauterine device” or “intrauterine devices” or iud*) NEAR/4 (medicated or hormonal or “hormone-releasing” or “progesterone-releasing”)
14. MeSH descriptor: [Ethinyl Estradiol] explode all trees
15. “ethinyl estradiol” or ethinylestradiol or “ethinyl oestradiol” or ethinyloestradiol
16. MeSH descriptor: [Desogestrel] this term only
17. Desogestrel
18. MeSH descriptor: [Levonorgestrel] this term only
19. Levonorgestrel
20. MeSH descriptor: [Norethindrone Acetate] this term only
21. “Norethindrone Acetate”
22. (progestin* or progesterone) NEAR/4 contracept*
23. “medroxyprogesterone acetate” NEAR/4 contracept*
24. (oral* or patch* or inject* or hormon* or medicated or postcoital or postcoitus or “post coital” or “post coitus”) NEAR/3 contracept*
25. estriol or oestriol
26. #9 or #10 or #11 or #12 or #13 or #14 or #15 or #16 or #17 or #18 or #19 or #20 or #21 or #22 or #23 or #24 or #25
27. #8 and #26

## Appendix B: Inclusion and exclusion criteria

The inclusion criteria are as follows:

- Cohort studies
- Cross-sectional studies
- Case-control studies
- Case series studies with >30 participants (for hypothesis purposes)
- Randomised controlled trials
- The outcome is MS
- The population must be women (aged 12 or above)\
- The exposure is prescribed HC medications
- Studies from any country or setting or language will be included
- Relative risk (RR), OR, incidence rate ratio and hazard ratio (HR) of MS as effect measures

The exclusion criteria are as follows:

- Reviews
- Case report studies
- Animal studies
- Case series studies with <30 participants
- Conference abstracts
- Population of men, or women under the age of 12
- Non-HC medications
- MS diagnosis following HC use, not before

## Appendix C: Data extraction

The following information was recorded for each study in the data extraction table:

- Study details (title, primary author, publication year)
- Geographical setting
- Primary outcome
- Study duration and follow-up period
- Number of participants (females only, mean/median age)
- Type of control group
- Number of women in control group
- Additional demographic factors of participants e.g. ethnicity (if available)
- Type of hormonal contraceptives
- Number of participants (%) who took each drug
- RR/ OR/ incidence rate ratio/ hazard ratio (crude and adjusted) per drug
- Statistical analysis
- Confounders adjusted for (e.g. body mass index, parity, family history, comorbidities, prior exposure to immunosuppressive drugs)
- Considered confounding by indication (Y/N)
- Clinical interpretation summary
